# Editorial: Cross-kingdom interactions in oral dysbiosis and host response

**DOI:** 10.3389/fdmed.2024.1398868

**Published:** 2024-03-25

**Authors:** Zvi G. Loewy

**Affiliations:** ^1^ Department of Pharmaceutical and Biomedical Sciences, Touro College of Pharmacy, Touro University, New York, NY, United States; ^2^ School of Medicine, New York Medical College, Valhalla, NY, United States

**Keywords:** microbiome, dysbiosis, oral care, periodontitis, halitosis

**Editorial on the Research Topic** Cross-kingdom interactions in oral dysbiosis and host response

Microbial dysbiosis is characterized by an imbalance of microbial species and a reduction in microbial diversity relative to the normal state of homeostasis. Dysbiosis results in a decrease of beneficial bacteria (commensal) and correspondingly an increase in bacteria that may be harmful (pathogens). The main factors influencing the composition of a microbiome that may cause dysbiosis include pharmaceuticals, specifically antibiotics, nutrition as well as psychological and physical stress (1, 2). Moderate shifts in microbial composition can enable additional factors to exacerbate an imbalance; these include increased oxidative stress, bacteriophages and bacteriocins (3).

The oral cavity is a complex environment populated by hundreds of diverse microbes. The oral microbial composition can evolve rapidly in response to oral environmental changes. The triggers for change in the oral microbiome encompass alterations in pH, nutrition and salivary fluid (4). When the composition and distribution of the oral microbes deviate from the norm due to dysbiosis, oral and systemic diseases result (5, 6). Oral diseases include caries and periodontal disease. Oral and systemic health links have been reported for cardiovascular problems, chronic obstructive pulmonary disease, inflammatory bowel disease and complications in pregnancy (7).

In this Research Topic, basic and review papers reported on the oral microbiome and dysbiosis and approaches to restore microbial homeostasis including innovative biomaterials designed to treat periodontal disease, the evaluation of oral care methods for removal of plaque demonstrated by digital imaging and qPCR analysis, characterizing the relationship of the oral microbiomes in halitosis and periodontitis and cell therapy as an approach for treating oral squamous cell carcinoma.

Current approaches for treating periodontal disease include tooth scaling and root planing or the use of photodynamic therapy (PDT). PDT is a non-invasive method that couples the use of a low-energy laser with a photosensitizer to eliminate pathogenic microorganisms. In a novel approach for treating periodontitis Zhu et al. report on the use of advanced biomaterials to facilitate periodontal regeneration. The authors described several different novel biomaterials designed to extend the release of antimicrobial agents and osteogenic molecules, or alternatively to enable healing by acting as immunomodulators.

Controlling oral plaque is fundamental to oral health. A clinical research study evaluating four methods for decreasing plaque was reported by Luo et al. The authors reported on a four arm clinical study: (1) A manual toothbrush group; (2) A manual toothbrush combined with an oral irrigator group; (3) An electronic toothbrush combined with an oral irrigator group; and (4) an electric toothbrush group. As measured by digital imaging and qPCR, dental plaque was found to decrease in all four groups. The electric toothbrush combined with an oral irrigator group demonstrated optimal plaque reduction.

An interesting review of the literature by Lee and Hong focused on addressing the hypothesis that the relationship between halitosis and periodontitis is mediated by the oral microbiome. Halitosis, the manifestation of unpleasant odors emanating from the oral cavity is attributed to volatile sulfur compounds (VSCs) produced by different microbes. Similar to halitosis, oral malodor is very prevalent in patients diagnosed with periodontitis. The authors proposed that unique oral microbiome profiles may be associated with halitosis and periodontitis. This suggests that specific microbes may be the root cause of the onset and progression of two oral diseases, halitosis and periodontitis. The authors conclude that animal studies and clinical studies are needed to validate the potential overlap and contribution of a specific oral microbiome to halitosis and periodontitis.

Summers et al. reviewed the process of chimeric antigen receptor T (CAR-T) cell therapy and the potential use in treating head and neck squamous cell carcinoma (HNSCC) by targeting the tumor associated antigens ErbB and MUC1 that are significantly expressed by HNSCC. The authors also reviewed combination therapies that use dual-targeting CAR-T cells and immune checkpoint inhibitors as well as CAR-T cells and NOTCH receptors.

Recurring themes in the current topic are: (1) The need to control and eradicate the microbial pathogens implicated in oral disease. As discussed, routine oral care procedures such as methods of brushing complexed with oral irrigation, as well as advanced novel biomaterials for delivering antimicrobials are of paramount importance in maintaining microbial homeostasis in the oral cavity; (2) Overlap of oral microbes implicated in different oral diseases requires additional studies; animal and human clinical investigations and (3) The role of oral microbial pathogens contributing to systemic disease and the need for developing and administering potent therapies including cell-based therapies requires additional investigation.

A positive impact on oral health predicated on controlling microbial dysbiosis will require future studies focused on biofilms, eradication of the biofilms and preventing cell dispersion. The dispersed cells when uncontained initiate biofilms elsewhere in the body leading to systemic disease. A systems approach that will encompass conventional oral healthcare methods coupled with novel antimicrobials, including those with anti-biofilm activity delivered by advanced biomaterials will deliver true oral and systemic health.
